# Selection platforms for directed evolution in synthetic biology

**DOI:** 10.1042/BST20160076

**Published:** 2016-08-15

**Authors:** Pedro A.G. Tizei, Eszter Csibra, Leticia Torres, Vitor B. Pinheiro

**Affiliations:** * Department of Structural and Molecular Biology, University College London, Gower Street, London, WC1E 6BT, U.K.; † Birkbeck, Department of Biological Sciences, University of London, Malet Street, WC1E 7HX, U.K.

**Keywords:** directed evolution, protein engineering, selection methodologies, synthetic biology, synthetic nucleic acid (XNA), xenobiology

## Abstract

Life on Earth is incredibly diverse. Yet, underneath that diversity, there are a number of constants and highly conserved processes: all life is based on DNA and RNA; the genetic code is universal; biology is limited to a small subset of potential chemistries. A vast amount of knowledge has been accrued through describing and characterizing enzymes, biological processes and organisms. Nevertheless, much remains to be understood about the natural world. One of the goals in Synthetic Biology is to recapitulate biological complexity from simple systems made from biological molecules–gaining a deeper understanding of life in the process. Directed evolution is a powerful tool in Synthetic Biology, able to bypass gaps in knowledge and capable of engineering even the most highly conserved biological processes. It encompasses a range of methodologies to create variation in a population and to select individual variants with the desired function–be it a ligand, enzyme, pathway or even whole organisms. Here, we present some of the basic frameworks that underpin all evolution platforms and review some of the recent contributions from directed evolution to synthetic biology, in particular methods that have been used to engineer the Central Dogma and the genetic code.

## What is directed evolution?

Directed evolution is a well-established approach for optimizing and engineering novel functions in both nucleic acids and proteins. It has been remarkably successful in isolating novel ligands and catalysts based on the natural biopolymers and it is an essential tool for exploring the potential of xenobiotic polymers–probing the boundary conditions of life itself. Directed evolution is usually compared with Darwinian selection because of similarities in their underlying principles: genetic diversity leading to diversity of phenotype, a link in selection between phenotype and genotype recovery and amplification of the selected genotypes.

Those simple principles hide a myriad of methodological details and caveats that must be considered in directed evolution experiments, including: library quality, evolutionary landscapes and evolvability, sequence spaces and selection methodologies. The only reasonable conclusion is that a range of tools are required to allow for flexibility in the evolution starting point (e.g. *de novo* compared with starting in the functional vicinity of the function sought), objective (e.g. from increased thermostability to the expansion of the substrate range beyond natural compounds), scale (e.g. from selection of a ligand to the evolution of an entire organism) and system (e.g. *in vitro* to a eukaryotic host).

Compared with rational design, a key advantage of directed evolution lies in the impact of knowledge gaps (or uncertainty). For rational design to be most effective, an accurate and complete understanding of the target system (the system to be modified) is required. Incomplete or incorrect understanding of the target system leads to a high failure rate of designs. Although failed designs can be used to improve understanding of the system being designed and of the design tools themselves–establishing a design, test, build and learn cycle characteristic of synthetic biology (see [1–4] for general reviews of synthetic biology)–this can be a lengthy and costly process.

On the other hand, evolutionary approaches can, at least in principle, bypass any knowledge requirements. Directed evolution relies only on a cycle of introducing diversity into a population followed by the partitioning of that population to isolate the desired function. Theoretically, any population can be systematically optimized towards the desired function by repeating cycles of directed evolution. In practice however, the number of variants in the population can rapidly escalate beyond the sampling capacity of any selection methodology. In addition, as a given functional variant becomes rarer in a population, there is a greater burden on the selection method to isolate them. Consequently, all available knowledge of the target system is considered when designing the directed evolution strategy to minimize the number of variants and maximize its likelihood of success.

The field of directed evolution has greatly expanded in the last 20 years and it would be impossible to discuss or even acknowledge all of our colleagues who have contributed to it in this brief review. We apologize to those whose work we have not been able to include and we highlight other excellent recent reviews covering different aspects of directed evolution, including library design and diversity creation [5–8].

Here, we focus on presenting a sample of the diverse array of selection methodologies developed to date and how they may be used to alter the core of biology: changing the genetic code, changing genetic materials, changing the very chemistry of life, as well as establishing independent systems that can coexist with nature (orthogonal systems). Modification of those processes critically change biological function and have been described as unnatural molecular biology [4] or xenobiology [9,10]. Beyond improving our understanding of life and its origins, these modifications can be applied to novel biocontainment strategies [9–11], therapeutic agents [12,13] and protein engineering [14].

## A strong phenotype–genotype linkage is at the heart of directed evolution

**Figure 1:**
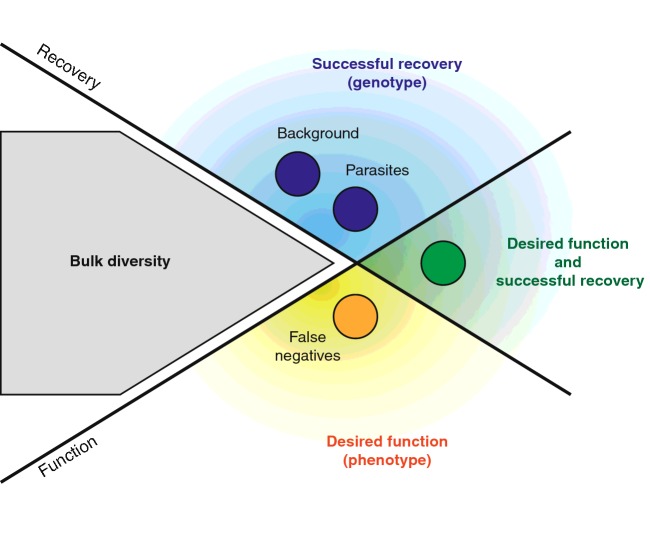
Principles of selection in directed evolution The goal of all selection (and screening) platforms is to partition a potentially large population (shown in grey as the bulk diversity) by function (phenotype) ensuring the recovery of the genetic information that accounts for that phenotype. Strong phenotype–genotype linkages allow efficient isolation of mutants with the desired function (green). Breakdown of that linkage results in false negatives (variants that have the desired function but that are not efficiently recovered–yellow) and false positives (variants that are recovered independently of the desired function–blue), which are integral aspects of all selection strategies.

Partitioning of a diversified population is, as mentioned above, a key part of the evolutionary process and the point at which the ‘fittest’ are selected, where fitness is used as a quantitative description of the ability of a particular variant to perform the function being selected. This process of separating variants can be done by serially probing individual variants (screening) or by probing populations in parallel (selection). Both partitioning strategies use a measure of function (phenotype) to separate the population in a way that allows recovery of the genetic information (genotype) that encodes for the function–establishing the phenotype–genotype linkage. Breakdown of that linkage results in false negatives and false positives that undermine the evolutionary process, as illustrated in Figure 1.

False negatives are variants with the desired function that fail to be recovered during selection, undermining the evolutionary process. The loss of such variants can occur at any step along the selection process: whether through poor expression (in the case of a protein) or lower stability of an enzyme variant, due to errors or high variation in the quantification of function, or through stochastic recovery in selection.

False positives, or variants that are recovered but that do not display the desired phenotype, are the result of two distinct processes: one random and non-specific (background), the other the result of a viable alternative phenotype that, although not desired, can be efficiently recovered (parasites). Background is usually generated by the partitioning process itself in selection, where non-specific interactions, e.g. DNA binding to nitrocellulose filters during aptamer selections [15], result in a sample of the population being recovered and taken forward to a subsequent selection round. By itself, background has little impact on the evolutionary process since further rounds of selection can be carried out until the desired function dominates the population. However, in methodologies prone to false negatives, the level of background recovery can have a significant impact on selection.

Parasites can inflict terminal damage to a directed evolution experiment by outperforming variants that display the desired phenotype–usually as a consequence of selection pressures rewarding phenotypes other than only the desired ones, or by parasitic variants outperforming the population during amplification (e.g. Spiegelman's Qβ serial dilution [16] or phage variants with high replication kinetics [17]). The impact of parasites in directed evolution is summarized by the maxim ‘you get what you select for’ that permeates the field [18].

The three populations (false negatives, parasites and background) are present in all selection and screening methods and this simple framework, shown in Figure 1, is a powerful tool to describe, analyse and design selection platforms–ensuring that selection pressures being used, maximize recovery of the desired mutants while taking steps to minimize or bypass the emergence of parasites.

Although a large number of selection platforms have been developed, they can be grouped into four categories: *in vivo*, *in vitro*, display systems (or *ex vivo*) and *in silico*. Each category has characteristic strengths and limitations that are the result of constraints imposed by where selection is carried out. *In silico* directed evolution, relying on computational tools to systematically generate, screen and optimize the biological function sought, is an emerging field with great potential but beyond the scope of this review [19–22].

The remaining three approaches have been remarkably successful at engineering individual biopolymers, gene circuits and genomes. In targeting core biological processes for modification, our knowledge and understanding of the systems being engineered is incomplete and limiting, making directed evolution an enabling technology and a powerful tool in the biologist's arsenal.

## *In vivo* directed evolution

**Figure 2:**
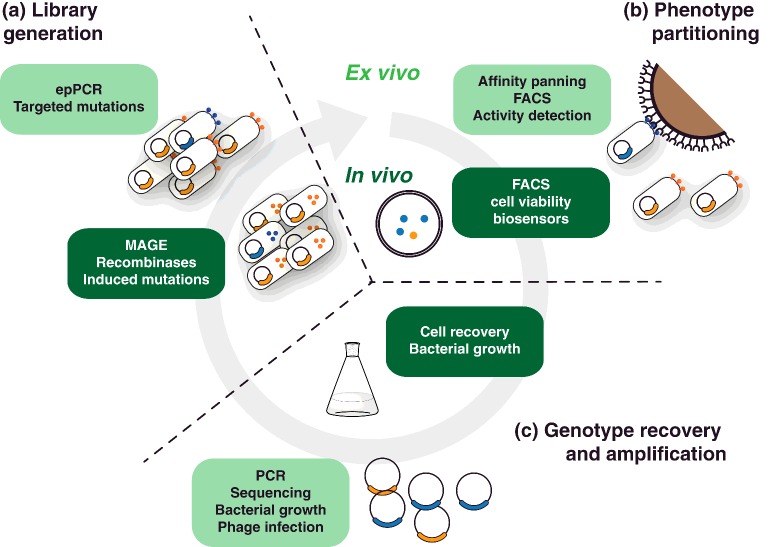
*In vivo* and *ex vivo* directed evolution Both strategies use the cell (or phage particle) as the physical linkage between genotype and phenotype through the directed evolution process. *Ex vivo* platforms tend to focus diversity (**a**) on to a single target gene, whereas *in vivo* platforms can extend that to metabolic pathways or even whole genomes. Once generated, the diverse repertoires are partitioned (**b**) with active (blue) variants preferentially recovered over inactive variants (orange). Partition by phenotype is linked to genotype recovery and amplification (**c**) which can take place in a single step if cells are still viable (as is the norm for *in vivo* methodologies). Alternatively, as shown for the *ex vivo* selection (light green boxes), genotype recovery and amplification can be separated, introducing different limitations to the process. The amplified recovered genotypes are the starting point of a subsequent round of selection.

Directed evolution using *in vivo* selection platforms is possibly the strategy closest to natural evolutionary processes: the cell itself provides the physical link between genotype and phenotype, imposing the constraint that the cell must remain intact and metabolically active during key stages of selection (Figure 2). Because the entire cellular genome can contribute to a phenotype, *in vivo* selection strategies can be particularly efficient at evolving complex phenotypes (or those that require multiple steps to be observed) with diversity targeted to particular genes of interest or potentially to the entire organism genome.

Traditionally, genomic mutations have been introduced stochastically through mutagenic compounds, stressors or by inducing a higher mutation rate in the host [23]. However, some strategies are available to improve targeting and efficiency of the mutagenic process. The multiplex automated genome evolution (MAGE) process developed by Wang, Isaacs and colleagues exploits the ability of the phage lambda Red system [24] to facilitate recombination of single stranded DNA oligos with bacterial genomes [25]. It was originally validated by introducing variation to 24 genetic components involved in the heterologous production of lycopene, generating a library of over 10^9^ variants partitioned by directly quantifying the synthesis of lycopene in colonies [25]. More recently, it has been used to systematically remove amber stop codons from the *Escherichia coli* genome [26,27], prior to re-introducing them to encode the incorporation of a non-canonical amino acid (ncaa) [26,27]–expanding the genetic code and creating a powerful approach towards the containment of genetically modified organisms.

Large-scale genome engineering can also be carried out by recombinases, which predictably and efficiently generate insertions, deletions and inversions. However, these enzymes usually require large (all longer than 30 base pairs) recognition sites that are naturally rare in the genome. Additional sites can be introduced in the genome, as is being done on an unprecedented scale in yeast [28–30], or by modifying recombinase target recognition [31,32].

As diversity is introduced in the system, selection begins and enrichment can be achieved by differences in cell survival, growth and replication rates or by partitioning the population based on the activity of a reporter gene (e.g. flow cytometric sorting using fluorescent reporters).

*In vivo* selection platforms have been successfully used in replacing natural amino acids for non-canonical ones, as well as in replacing natural nucleobases with unnatural ones in a bacterial genome. Serial passaging, in which a growing culture is diluted at an arbitrary cell density, has been successfully used by Budisa and colleagues to completely replace L-tryptophan with L-β-(thieno[3,2-b]pyrrolyl)alanine in the *E. coli* proteome [33]. Turbidostats, automated platforms that regularly adjust the rate of culture dilution based on culture density, have also been successfully used to adapt organisms to grow in substrates not naturally used in the starting organism. Using a dual turbidostat platform, Mutzel and colleagues [34] systematically evolved thymine auxotrophic *E. coli* strains that could thrive on chlorouracil, replacing thymine in its genetic material–a demonstration that genetic information storage itself can be changed if sufficiently small steps can be taken. The dual turbidostat effectively avoided biofilm-forming variants, a known parasite in continuous culture methods, but the method was still vulnerable to parasites that could synthesize thymine through uncharacterized salvage pathways, potentially reducing the selective pressure of the system. Indeed, Mutzel and colleagues identified a novel salvage pathway through tRNA U54 methyl transferase (*trmA*) that could provide up to 10% of the genome's thymidine. Deletion of *trmA*, reduced the remaining genomic thymidine levels to below 1.5%, the limit of detection in that particular approach.

Another approach to reduce the emergence of parasites is by combining selection strategies that differ in their vulnerabilities to parasites, such as those commonly used in the directed evolution of orthogonal aminoacyl tRNA synthetases (aaRSs) [35,36]. Orthogonality between endogenous aaRSs/tRNAs and the aaRS/tRNA pair being introduced is essential for the efficient, site-specific incorporation of ncaa, and has been successfully obtained by a combination of positive and negative selection steps. Positive selection requires ncaa incorporation at an amber stop codon placed in a selectable marker gene (e.g. antibiotic resistance marker) to ensure survival. A second selection step, a negative selection, omits the ncaa and requires that no other amino acid is incorporated at the same codon in a gene coding for a toxic product (e.g. a nuclease), which would lead to cell death [36]. Parasites that may bypass the positive selection by allowing the misincorporation of any natural amino acid are penalized in the negative selection. Similarly, inactive variants that may become parasites of the negative selection are then penalized in the positive selection–thus increasing the power of the methodology.

Biological logic circuits can also be implemented to enhance *in vivo* selection platforms, either by monitoring the overall state of the cell [37] or by responding to one or multiple inputs relevant to the selection process. These circuits can be single component circuits as recently reported by Baker and colleagues for the *in vivo* selection of progesterone synthesis in yeast [38]. The progesterone biosensor was the fusion of an unstable domain to a reporter or selectable marker: progesterone binding stabilizes the protein allowing reporter expression–fulfilling its biosensor role.

A more complex circuit was developed by Chou and Keasling for the optimization of lycopene production [39]. By coupling detection of a lycopene biosynthetic intermediate with DNA polymerase III repression, the circuit could couple low lycopene production with the host mutation rate. Increasing levels of lycopene, detected by the red colour of isolated colonies was used to guide selection towards higher producers in repeated rounds of selection.

Logic circuits do not need to be directly linked to the phenotype under selection. Ellington and colleagues [40] established a selection platform, compartmentalized partnered replication (CPR), based on a logic circuit in which the output is the expression of the *Thermus aquaticus* thermostable DNA polymerase (*Taq*). After selection, cells are compartmentalized and successful circuits use the expressed *Taq* to amplify the genes of interest in an emulsion PCR reminiscent of compartmentalized self-replication [41]. As with other techniques that use PCR to amplify selected information, directed evolution is confined to the region bound by the final PCR.

## *In vitro* directed evolution

*In vitro* directed evolution is characterized by the use of selection platforms that either bypass living cells entirely (a fully *in vitro* system) or that rely on living hosts simply for the maintenance or heterologous expression of the biopolymers to be selected (a partially *in vitro* platform). It can be used to isolate biopolymers that would otherwise be toxic to a host or that function in conditions incompatible with biology, such as in the presence of denaturants, solvents or extreme temperatures. By carrying out selection outside biology, at least some of constraints of *in vivo* selection platforms can be bypassed, such as toxicity and bottlenecks in host transformation. Fully *in vitro* systems allow cells to be bypassed altogether, and with them the limit of transformation efficiency and recovery (typically ∼ 10^8^ CFU (colony forming units) per transformation), enabling libraries of 10^14^ to be achieved and increasing the available sequence search space.

*In vitro* selection allows us to explore and potentially recapitulate processes thought to have occurred early in life–abiotic processes in which a single molecule retains both genotype and phenotype–through the directed evolution of nucleic acid ligands (aptamers–recent review here [42]) and enzymes (NAzymes). Systematic evolution of ligands by exponential enrichment (SELEX) [43] is well established allowing not only directed evolution of functional RNA molecules [44–46], but also functional DNA [47–49] and synthetic nucleic acids (XNAs) [50–53]. Notably, SELEX is a potential misnomer because initial libraries are sufficiently large as to include all possible variants. Since it remains technically difficult to prove an isolated functional molecule was not present in the starting library, isolation of a functional nucleic acid can be the result of selection only - and not directed evolution since no evolution would be required (where possible that distinction is maintained in the text). In most cases, selection involves isolating functional nucleic acids, converting them to DNA (to allow efficient amplification by PCR), and the regeneration of the functional nucleic acid repertoire for further rounds of selection.

Variations of SELEX allow the RNA or XNA reverse transcription to DNA to be bypassed, enabling functional nucleic acids based on chemistries that are not viable genetic materials (or for which no efficient reverse transcriptase is known). In these DNA display methodologies, the genotype (encoded in the DNA) remains physically attached to the functional nucleic acid. Recovery of functional molecules therefore retrieves the encoding DNA–the important phenotype–genotype linkage.

Liu and colleagues have extended DNA display technology to enable selection of small molecules and peptide nucleic acids *in vitro* [54–56] and even clusters of carbohydrates [57,58]. In addition, DNA display methods have also been developed for the evolution of proteins, particularly nucleic acid processing enzymes where function can be linked to binding or modification of the relevant genotype [59–62]. This can be extended to other functions but generally rely on fusions that enable a covalent link between enzyme being tested and its genotype [63].

Genotype display, however, need not imply DNA, with alternatives developed for linking mRNA (genotype) to function (phenotype). In mRNA display platforms, a covalent link is made between the protein (selected for phenotype) and its encoding mRNA (genotype) during *in vitro* translation [64]. This platform has been widely used for the isolation of protein binders from *in vitro* translated protein libraries, including selecting for *de novo* functionality, such as a protein capable of ATP binding [65]. Seelig and Szostak [66,67] demonstrated that an RNA ligase could be obtained, by mRNA display, from a naïve library without prior knowledge of mechanism or sequence landscape–highlighting the power of directed evolution. mRNA display has also been combined with the incorporation of unnatural amino acids and click chemistry to create libraries of glycopeptides, containing multiple Man_9_ glycans bound to a translated peptide. Variants capable of binding with high affinity to the HIV neutralizing antibody 2G12 have been efficiently selected by this method with possible relevance for HIV vaccine development [68].

It is also possible to link protein and mRNA non-covalently, such as in ribosome display–where a stalled ribosome works as an adaptor, linking mRNA to the displayed protein. Ribosome display has been successfully applied to the selection of ligands to different molecular targets [69–72], and more recently to the selection of catalysts [73–75].

An alternative strategy for *in vitro* selection is to co-isolate genotype and phenotype in cell-like compartments, termed *in vitro* compartmentalization (IVC), through the use of emulsions. Typically, water-in-oil emulsions do not allow significant exchange of components and hence are used to isolate genotype and phenotype inside individual compartments, ensuring a robust phenotype–genotype linkage [76]. Notably, compartmentalization can be achieved through other means, such as eutectic phases [77].

A number of emulsions have been explored for directed evolution with some stable even in extreme reaction conditions, such as the high temperatures required for emulsion PCR and for the selection of thermostable enzymes [41,76]. Emulsions can be made in bulk [76,78], resulting in a polydisperse emulsion (where compartments have a distribution of sizes), or in microfluidic devices where compartment size can be tightly regulated–a monodisperse emulsion [79]. Compartment size variation can impose experimental constraints: bulk emulsions, requiring less specialized equipment, are easier and faster to make than an equivalent emulsion made with microfluidic systems. However, compartment size variation may affect platforms where selection is carried out near the *K*_D_ of the target enzyme, and where the signal generated in a compartment is used to partition the population, since reporter signals can depend on compartment size and concentration [80]–these can undermine platforms using bulk emulsion with higher rates of false negatives and false positives.

Emulsions have become the basis of several selection and screening strategies. Microfluidics platforms have been developed to introduce single cells (or beads) per compartment [81,82] with compartments being individually sorted [83–85], fused, split, stored and disrupted [86]–providing a more versatile range of methods than it is possible in bulk emulsions. For instance, Chaput and co-workers developed a microfluidic-based platform for the selection of polymerases that employs an optical detection of the enzymatic activity followed by fluorescence-associated cell sorting (FACS) enrichment [87].

Holliger and colleagues, in developing compartmentalized bead tagging (CBT) [88], demonstrated that it is possible to change the contents of the compartments in a bulk emulsion. In CBT, ribozyme genes were bound to paramagnetic beads and transcribed *in vitro* in a first emulsion. The transcribed ribozymes were ligated to the bead, allowing the first emulsion to be disrupted without breaking the phenotype–genotype linkage (between gene and ribozyme). Recovered beads could then be re-emulsified for ribozyme selection in a second bulk emulsion.

In addition to recapitulating processes from early biology, such as RNAzyme-based RNA replication, *in vitro* selection platforms have also been used to expand the Central Dogma. Holliger and colleagues, used an IVC selection strategy termed compartmentalized self-tagging (CST) to isolate thermostable DNA polymerase variants capable of synthesizing a number of different XNAs [50]. Together with a rationally designed reverse transcriptase, this demonstrated that the natural nucleic acids are not unique in being able to store genetic information. Although the polymerases isolated could synthesize XNAs, they retained DNA polymerase activity limiting their use towards introducing XNAs *in vivo*.

**Figure 3:**
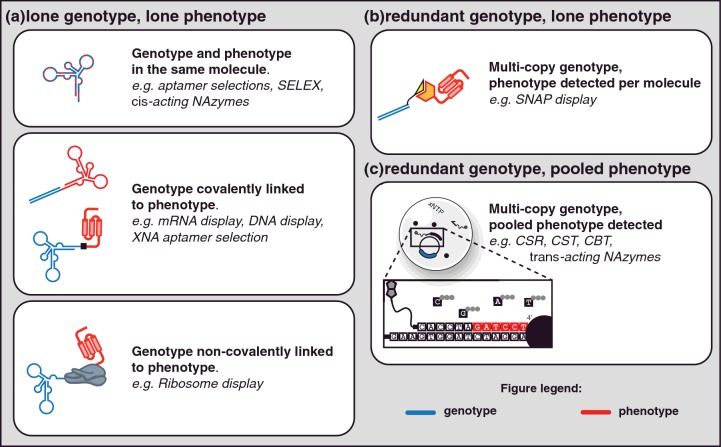
*In vitro* selection Platforms for *in vitro* selection can be broadly divided by the available redundancy of phenotype and genotype linkages. In a number of selection strategies, the link is unique–a lone genotype molecule is linked to a lone molecule that may have the phenotype being selected (**a**). Compartmentalization strategies enable redundancy in the system with one-to-many [redundant genotype to lone phenotype (**b**) or lone genotype to pooled phenotype (not shown)] and many-to-many [redundant genotype to pooled phenotype (**c**)] mappings between phenotype and genotype available.

Compartmentalized systems have few but significant advantages over other *in vitro* selection platforms. The added compartmentalization minimizes cross-reactivity (or cross-catalysis) that can negatively affect *in vitro* platforms creating phenotype–genotype linkages on inactive variants. In addition, compartmentalization allows for changes in the topology of selection that make the platform more robust (Figure 3). For instance, in ribosome display a single mRNA molecule is linked to a single protein (a one-to-one mapping). Any RNA degradation or amino acid misincorporation destroys the phenotype–genotype linkage of that molecule (creating a false negative). On the other hand, in compartmentalized systems, multiple enzymes can act on multiple substrates to create a robust phenotype–genotype linkage (a many-to-many mapping) such that degradation of one substrate, catalyst or one molecule of the genotype does not undermine the phenotype–genotype linkage. Robustness in one-to-one platforms is achieved through redundancy in the initial library whereas a many-to-many selection strategy can achieve a significant level of robustness even in low redundancy repertoires.

## *Ex vivo* directed evolution

*Ex vivo* selection, more commonly referred to as surface display, groups platforms and methodologies in which the biopolymer under selection (usually a protein) is accessible (i.e. outside) but still attached to a host, be it a bacteriophage or whole cell (Figure 2). Like *in vivo* platforms, a key advantage of *ex vivo* systems is that selection can be carried out using live hosts (or infective viable phages), bypassing costly intermediate steps and streamlining the evolution process. Crucially, *ex vivo* platforms bypass the challenge of bringing reagents (or targets) into the host, and extend the reaction conditions available for selection; though the latter is still limited to conditions that do not disrupt the host or the link between displayed biopolymer and host, both of which would undermine the phenotype–genotype linkage.

Phage display, in which gene fusions allow the display of a protein on the surface of a phage particle, is the earliest and by far the most successful *ex vivo* platform developed to date [89]. It has been extensively used in the development of antibody-based therapeutics [90] as well as for isolating a range of other ligands and enzymes [90–93].

Being a mature technology has allowed researchers to probe its shortcomings and biases in a series of carefully controlled experiments [17] showing, for instance, that abundance does not correlate with binding affinity in phage display selections. This can be rationalized by conceptualizing partitioning as the result of two selective processes: binding and amplification [17]. The latter is also effectively a selection step, and isolated variants with low amplification kinetics will be selectively lost (false negatives). A subsequent deep sequencing analysis quantified the diversity drop from a 10^6^ library to enrichment of ∼150 clones, which dominated 20% of the selected library [94]. Some of those biases can be avoided by the use of emulsions or droplet PCR [95–97] or careful functional variant identification (via deep sequencing, [94]). Further, the use of bioinformatics analyses or selection databases [98–100] may allow the exclusion of parasites. Akin to the development of orthogonal aaRSs by multiple selections that differ in how they can be exploited by parasites, such problems can be circumvented by the use of different hosts (e.g. M13 compared with lambda) for each round of selection.

Currently, bar a few exceptions, surface display methods remain an under-exploited technology in the directed evolution toolbox for synthetic biology. For instance, phage display has been used to engineer thermostable DNA polymerases with extended substrate spectra [101–103], novel ligands using expanded genetic codes [104] and even to test the impact of the correlation between chemical diversity in the genetic code and fitness [105]. Key to those developments is the *ex vivo* localization of the biopolymers being selected, which enables their access to non-biological materials (e.g. xNTPs, oligonucleotides).

*Ex vivo* selection, however, extends much further than bacteriophages with display platforms established for Gram-negative and Gram-positive bacteria [106–108], as well as alternative platforms such as display on the surface of *Bacillus subtilis* spores [109]. Display platforms in eukaryotic cells, both yeast and cultured mammalian cells [110–112] have also been demonstrated and are of particular interest as they allow the incorporation of post-translational modifications in the passenger, particularly relevant to therapeutic antibody engineering.

Common to current *ex vivo* platforms is that display is achieved through gene fusion between a protein that naturally localizes to the surface of the host and the protein of interest (or passenger). This imposes some of the key constraints of the technology: the protein of interest and its fusion partner have to remain active once fused and be successfully exported to the host surface. For instance, export to the host surface is generally not an issue in display platforms based on lytic bacteriophages (e.g. T7), as the phage capsid is synthesized and assembled in the bacterial cytoplasm where most proteins fold efficiently. However, the fused host-selection protein cannot significantly affect the capsid assembly process or the function of the capsid in virulence; the latter is a constraint if phages are being recovered by infection of a susceptible host.

The use of cells, or other sizeable particles such as liposomes or other double emulsions [113,114] and beads [88,115,116], enables the partitioning of the population by FACS [90,117]. Although FACS is a high-throughput screening tool rather than selection, it provides an unparalleled level of flexibility, allowing display levels to be normalized and quantification of multiple parameters of a population. It also enables methods for coupling enzyme function to fluorescence. A particularly powerful method was developed recently in which hydrolase function on the cell surface is linked to the covalent attachment of biotinylated tyramide via horseradish peroxidase [118,119]. The success of this technique to quantitatively couple catalytic activity to fluorescent labelling for evolution [120] suggests that tools such as this one will be invaluable to allow the more widespread adoption of cell display for enzyme engineering for a wider range of functionalities [121].

## Directed evolution as a tool for synthetic biology

Despite the diversity and versatility of selection platforms available, novel ones are regularly being developed–delivering custom solutions to ever growing challenges. As molecular biology methods and technologies develop, novel strategies to diversify and partition a biopolymer population become available, increasing experimental control, throughput and pace.

Directed evolution performs the design, build and test cycle of synthetic biology on a scale that is unnatural in engineering: it would be the equivalent of building millions (or even trillions) of slightly different machines (e.g. watches) in search of a specific improvement (e.g. more precise time keeping). On an engineering scale, such approach would be prohibitive, if even possible. However, on a biological scale, millions are still small numbers, barely able to cover the immediate sequence neighbourhood of even a small protein.

Directed evolution has successfully been used to isolate novel and optimize existing function on natural and synthetic biopolymers. But its key strength lies on how it deals with uncertainty. Even in the absence of complete understanding of complex biological systems, directed evolution is a powerful tool to re-engineer even the most central truths of life on our planet–that life is based on DNA and RNA, and that life requires (or is optimal with only) 20 amino acids.

## Abbreviations

aaRS: aminoacyl tRNA synthetase
CFU: colony forming units
FACS: fluorescence-associated cell sorting
IVC: *in vitro* compartmentalization
ncaa: non-canonical amino acid
SELEX: systematic evolution of ligands by exponential enrichment
XNA: synthetic nucleic acid
